# A Tool for Interactive Data Visualization: Application to Over 10,000 Brain Imaging and Phantom MRI Data Sets

**DOI:** 10.3389/fninf.2016.00009

**Published:** 2016-03-15

**Authors:** Sandeep R. Panta, Runtang Wang, Jill Fries, Ravi Kalyanam, Nicole Speer, Marie Banich, Kent Kiehl, Margaret King, Michael Milham, Tor D. Wager, Jessica A. Turner, Sergey M. Plis, Vince D. Calhoun

**Affiliations:** ^1^The Mind Research Network and Lovelace Biomedical and Environmental Research InstituteAlbuquerque, NM, USA; ^2^Intermountain Neuroimaging Consortium, University of Boulder ColoradoBoulder, CO, USA; ^3^Department of Psychology, University of New MexicoAlbuquerque, NM, USA; ^4^The Child Mind Institute and The Nathan Kline InstituteNew York, NY, USA; ^5^Department of Psychology, Georgia Tech UniversityAtlanta, GA, USA; ^6^Department of Electrical & Computer Engineering, University of New MexicoAlbuquerque, NM, USA

**Keywords:** data sharing, big data, neuroscience, magnetic resonance imaging (MRI), t-SNE (t-distributed stochastic neighbor embedding), visualization

## Abstract

In this paper we propose a web-based approach for quick visualization of big data from brain magnetic resonance imaging (MRI) scans using a combination of an automated image capture and processing system, nonlinear embedding, and interactive data visualization tools. We draw upon thousands of MRI scans captured via the COllaborative Imaging and Neuroinformatics Suite (COINS). We then interface the output of several analysis pipelines based on structural and functional data to a t-distributed stochastic neighbor embedding (t-SNE) algorithm which reduces the number of dimensions for each scan in the input data set to two dimensions while preserving the local structure of data sets. Finally, we interactively display the output of this approach via a web-page, based on data driven documents (D3) JavaScript library. Two distinct approaches were used to visualize the data. In the first approach, we computed multiple quality control (QC) values from pre-processed data, which were used as inputs to the t-SNE algorithm. This approach helps in assessing the quality of each data set relative to others. In the second case, computed variables of interest (e.g., brain volume or voxel values from segmented gray matter images) were used as inputs to the t-SNE algorithm. This approach helps in identifying interesting patterns in the data sets. We demonstrate these approaches using multiple examples from over 10,000 data sets including (1) quality control measures calculated from phantom data over time, (2) quality control data from human functional MRI data across various studies, scanners, sites, (3) volumetric and density measures from human structural MRI data across various studies, scanners and sites. Results from (1) and (2) show the potential of our approach to combine t-SNE data reduction with interactive color coding of variables of interest to quickly identify visually unique clusters of data (i.e., data sets with poor QC, clustering of data by site) quickly. Results from (3) demonstrate interesting patterns of gray matter and volume, and evaluate how they map onto variables including scanners, age, and gender. In sum, the proposed approach allows researchers to rapidly identify and extract meaningful information from big data sets. Such tools are becoming increasingly important as datasets grow larger.

## Introduction

Visualizing high-dimensional data in a quick and simple way to produce meaningful information is a major challenge in the field of big data. This is particularly true for the neuroimaging community, where researchers commonly rely on the visual inspection of individual images as their primary means of quality control (QC). Although reasonable for smaller sample sizes, inspection strategies are limited in their ability to identify meaningful patterns in large data sets or to update their strategies as new data are added. Additionally, as technological innovations, such as multiband MRI pulse sequences (Van Essen et al., 2012) increase the size and dimensions of the datasets obtained, it becomes harder to extract meaningful information due to the complexity and size of the data. Perhaps most challenging, are data aggregation initiatives attempting to pool data across multiple imaging sites—especially when the data is heterogeneous (i.e., data collection protocols differ across sites; Potkin and Ford, 2009; Van Horn and Toga, 2009; Consortium, 2012; Di Martino et al., 2013). These challenges working with high dimensional data sets make simple and efficient quality control and information extraction from brain imaging data quite demanding.

Here, we propose a new web-based approach based on the use of a nonlinear embedding algorithm called t-distributed stochastic neighbor embedding (t-SNE; van der Maaten and Hinton, 2008) and data driven documents (D3)^1^ JavaScript for rapid visualization of large MRI datasets. The input dataset is passed through the dimension reducing t-SNE algorithm to create a 2-dimensional (2D) data set while preserving local relationships. The resulting 2D plot is then visualized on a web page as 2D interactive scatter plots using D3 JavaScript. Each point in the plots represents a scan. D3 JavaScript allows us to show relevant information about each scan on the plots in real time to help identify the reason behind the grouping of the data. This visualization tool provides a way for researchers to quickly identify interesting patterns in the data with respect to differences across scanners, sites, scanning protocols, scan collection times, studies, demographic information, and more.

Note, we are not proposing an automated clustering/decision making approach in this work, rather our goal is to demonstrate the utility of combining a data reduction approach with interactive visualization/color coding to provide a way for users to identify interesting patterns within extremely large data sets. Once these patterns are identified, a deeper investigation of the respective data sets could reveal more detailed useful information (as for example we show for some of the data sets in this paper). Such an approach does not replace standard QC approaches, but we contend that additional value is added by providing tools to provide users with a high-level view of their data. As we show, such a view can reveal information that is not detected with standard QC and also provide a useful exploratory tool to interactively identify how variables of interest are encoded within the data or to assess how similar newly collected data are to existing data sets.

Amongst the existing dimensionality reduction algorithms, t-SNE effectively minimizes the problem of cost function optimization and also addresses the “crowding problem,” i.e., it produces significantly better visualizations by reducing the tendency to crowd points together in the center of the map. T-SNE enables us to create a single map that integrates structure at many different scales. This is particularly important for high-dimensional data that lie on several different, but related, low-dimensional manifolds (or sets), such as images of objects from multiple classes seen from multiple viewpoints, especially human brain MRI scans (van der Maaten and Hinton, 2008). This paper introduces the concept of interactive visualization by using various types of datasets as shown in Table 1. These data sets include phantom data, human structural data, and human functional data from multiple sites and scanners. Next, we highlight several use cases where this tool can be of use to understand trends/patterns in the data. Finally, we discuss the results and also some of the many possible extensions of this idea (including other algorithms, different metrics, and more).

**Table 1:** **Overview of various data and experiments**.

**Type of data**	**Size of input dataset**	**t-SNE inputs**	**Number of dimensions of input dataset**	**Number of Dimensions after running PCA**	**t-SNE learning rate**	**t-SNE perplexity**
Phantom	277	Pre-processed QC values	4	4	500	30
fMRI	3910	Pre-processed QC values	6	6	1000	30
sMRI-1 (Pre-processed data from FreeSurfer pipeline)	2806	Free Surfer volumetric measurements of various brain regions	65	50	1000	30
sMRI-2 (Pre-processed gray matter images from VBM pipeline)	9963	Voxel values from gray matter segmentation images	9919	50	1000	20
ABIDE fMRI	1153	Pre-processed QC values	22	22	1000	30
Software				Matlab Version 7.12.0.635 (R2011a) 64-bit (glnxa64)		
Operating system				Ubuntu Version 12.04		
Processor				Intel(R) Xeon(R) CPU E7-4870		
Processor speed				2.4 GHz		
Number of CPU cores				10		
Number of Threads				20		
Cache size				30 MB		
RAM				512 GB		

The t-SNE approach has been used successfully in previous work to provide data visualization between patients and controls (Ridgway et al., 2012) or in the context of structural MRI and diffusion weighted imaging data to visualize males vs. females (Mwangi et al., 2014). Both of these examples used data from less than 100 subjects and were focused on a specific problem which showed promise. In this work we expand to multiple additional examples including phantom data and various levels of preprocessing, including data from over 10,000 data sets in addition to proposing an interactive visualization approach which can be used to evaluate new data on the fly.

## Methods and data

We demonstrate our approaches with the following use cases: (1) quality control measures calculated from phantom data, (2) quality control metrics computed from human functional MRI data across various studies, scanners, sites, (3) volumetric measures from human structural MRI data across various studies, scanners and sites, (4) gray matter density values from all brain voxels. We leverage thousands of datasets including phantom, human brain structure, and human brain function datasets captured via an existing neuroinformatics data capture and management system called the COllaborative Imaging and Neuroinformatics Suite (COINS; http://coins.mrn.org; Scott et al., 2011; King et al., 2014; Wood et al., 2014) to demonstrate the potential of the proposed visualization technique (more details on COINS can be found in Appendix A). Written consent was obtained from all subjects according to institutional guidelines at the University of New Mexico Human Research Protections Office or The Colorado University-Boulder's IRB Office and all data were anonymized prior to group analysis. An automated analysis pipeline is run on the data, producing a preprocessed data set, which is then processed via the t-SNE algorithm. The t-SNE algorithm reduces the number of dimensions for each scan in the input data set to two, using principal component analysis (PCA) and a nonlinear dimensionality reduction technique, while preserving the local structure of data sets (van der Maaten and Hinton, 2008). Based on the specific use case, different data can be provided as input. The number of dimensions of the input dataset is the number of inputs passed to the t-SNE, i.e., QC metrics, brain volumes, or voxel values in the examples we provide.

Two distinct approaches were used to visualize the data. In the first approach, we computed multiple summary QC values from the data and used these as inputs to the t-SNE algorithm. In the second case, computed variables of interest (e.g., brain volume), and the voxel values from the pre-processed gray matter images from complete brain were used as inputs to the t-SNE algorithm. These approaches are by no means exhaustive; rather, the metrics outlined in this paper are only a few of many possible metrics, chosen to demonstrate the potential of the proposed visualization technique. Nonetheless, the t-SNE inputs used in the proposed technique have proven to be robust, in terms of extracting meaningful information from data across various sites and studies.

**Figure A4:**
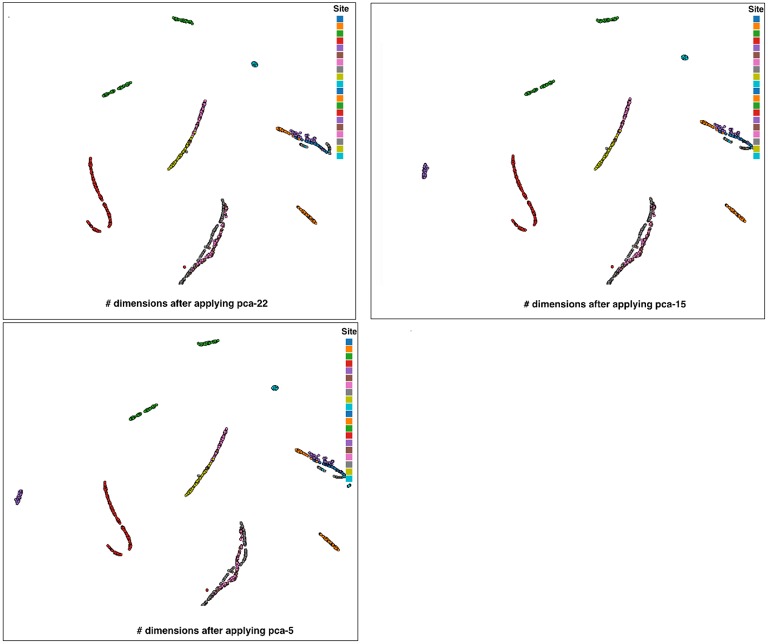
**ABIDE fMRI PCA experiments**. Plots are color coded by site. Note, the X and Y coordinate on the plot are not labels as they are arbitrary and simply represent the two dimensions of the t-SNE result, one can infer that if two points are closer together in the 2D graph their data are more similar to one another as quantified by the t-SNE criteria mentioned above.

**Figure A5:**
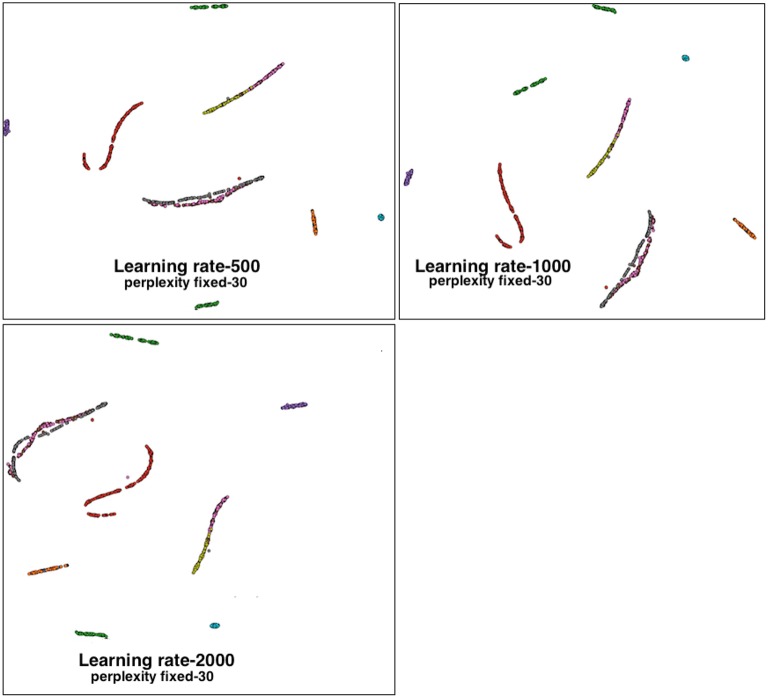
**ABIDE fMRI Learning rate experiments**. Plots are color coded by site. Note, the X and Y coordinate on the plot are not labels as they are arbitrary and simply represent the two dimensions of the t-SNE result, one can infer that if two points are closer together in the 2D graph their data are more similar to one another as quantified by the t-SNE criteria mentioned above.

**Figure A6:**
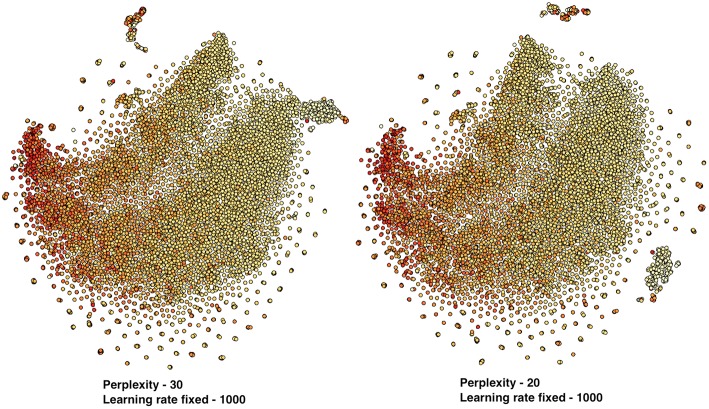
**sMRI-2 perplexity experiments**. Plots are color coded by age. Note, the X and Y coordinate on the plot are not labels as they are arbitrary and simply represent the two dimensions of the t-SNE result, one can infer that if two points are closer together in the 2D graph their data are more similar to one another as quantified by the t-SNE criteria mentioned above.

Table 1 shows the summary of various datasets used and the respective experimental setup. We indicate the size of the input dataset in subjects, while the t-SNE learning rate and t-SNE perplexity are dimensionless. Note, that PCA was not run on the phantom and fMRI QC datasets for our initial use cases since the input dimension was small. ABIDE fMRI data sets were evaluated with several PCA dimensions including 15 and 5, and the overall results were robust to the choice of PCA dimension. These results are shown in Figure A4. The sMRI data sets were reduced somewhat, and for these we also evaluated higher PCA dimensions including 100 and 150 and found our conclusions were similar regardless of the specific value (though more testing could be done for this in future work). This is also generally true of the other two parameters (perplexity and learning rate). Learning rate controls the rate of change of the algorithm parameters during the learning process. For learning rate, the default parameter was 500, we increase this value for the more complex data sets, but the results were similar. Some of the results using various learning rate values on ABIDE fMRI data set are shown in Figure A5. Perplexity is a measure for information, defined as 2 to the power of the Shannon entropy. The perplexity of a fair die with k sides is equal to k. In t-SNE, the perplexity influences the number of effective nearest neighbors. The performance of t-SNE is fairly robust under different settings of the perplexity. Some of the results using various perplexity values on sMRI-2 data set are shown in Figure A6. Typical values for the perplexity range between 5 and 50. Other default values of input parameters of t-SNE are outlined in Appendix B. Appendix C shows detailed summary of the input datasets with respect to scanner type and studies.

### t-SNE nonlinear embedding

For the t-SNE algorithm each data set is first converted to a matrix of N rows and M columns, where N is the number of datasets and M is the number of dimensions of each dataset. For example, if we have 10 scans and each scan has 60 QC measures, then the size of the matrix is 10 rows and 60 columns. We first apply PCA on this dataset to reduce the dimensionality of the data. This speeds up the computation of pairwise distances between the data points and suppresses noise without severely distorting the interpoint distances. Then a nonlinear dimensionality reduction algorithm is used to convert the PCA-reduced representation to a two-dimensional map. The resulting map is displayed as a scatterplot. The X and Y axis in all the scatterplots are the values of the two dimensions from the t-SNE output. Depending on the modality, the correlation between dimensions of the input dataset and the type of data (e.g., phantom data, structural MRI, functional MRI), the values of various parameters in the t-SNE algorithm are manually tuned but the specific value did not have a huge impact on the overall pattern observed (see Table 1). The accuracy of the grouping of datasets from results of this algorithm is verified by manual examination of the scans.

We briefly review the t-SNE algorithm as follows: The input data set is *X* = {*x*_1_, *x*_2_, *x*_3_, …, *x*_*n*_} and the resulting low-dimensional data is represented as $Y^{T}=\;\left\{y_{1},\;y_{2},\;y_{3},\ldots ,y_{n}\right\}$. Stochastic neighbor embedding (SNE) first converts the high-dimensional Euclidean distances between data points into conditional probabilities that represent similarities. The similarity of data point *x*_*j*_ to datapoint *x*_*i*_ can be computed as a conditional probability, *p*_*j*|*i*_, such that *x*_*i*_ would pick *x*_*j*_ as its neighbor if neighbors were picked in proportion to their probability density under a Gaussian centered at *x*_*i*_ with standard deviation _*i*_. For nearby data points, *p*_*j*|*i*_ is relatively high, whereas for widely separated data points, *p*_*j*|*i*_ will be almost infinitesimal (for reasonable values of σ*i*). We can compute a similar probability, *q*_*i*_, based on the output data. The perplexity is defined as $Perp\left(P_{i}\right)=2^{H\left(P_{i}\right)}$ where *H*(*P*_*i*_) is the Shannon entropy of *P*_*i*_. Based on the above, a cost function *C*, the Kullback-Leibler divergence between *P* and the Student-t based joint probability distribution *Q*, where *P* and *Q* represent the afore mentioned conditional probabilities over all other data points or map points, can be computed and optimized via a gradient descent method as $\frac{\delta C}{\delta y_{i}}=4\sum_{j}\left(p_{ij}-q_{ij}\right)\left(y_{i}-y_{j}\right){\left(1+{\left\|y_{i}-y_{j}\right\|}^{2}\right)}^{-1}$. The basic algorithm is as follows (van der Maaten and Hinton, 2008):

begin

           compute pairwise affinities *p*_*j*|*i*_ with perplexity *Perp* (as defined above)

           set *p*_*ij*_ = (*p*_*j*|*i*_ + *p*_*i*|*j*_)∕(2*n*)

           sample initial solution $y^{\left(0\right)}=\left\{y_{1},y_{2},\ldots ,y_{n}\right)$ *from N* (0, 10^−4^*I*)

           **for** *t*=1 **to** *T* **do**

              compute low-dimensional affinities *q*_*ij*_ (as defined above)

              compute gradient δ*C*∕δ*y* (as defined above)

           set $y^{\left(t\right)}=y^{\left(t-1\right)}+\eta \left(\frac{\delta C}{\delta y}\right)+\alpha \left(t\right)\left(y^{\left(t-1\right)}-y^{\left(t-2\right)}\right)$

      end

end

There are multiple parameters that need to be selected including the cost function parameter perplexity and the optimization parameters the number of iterations *T*, the learning rate η, and the momentum α(*t*). The optimization of these parameters for a specific application domain is needed, though in our initial evaluations the results were relatively robust to changes in the parameters. It may be useful for the user to tweak these as another aid in the visualization of the data, thus in our implementation we provide access to these parameters for the user. Additional information on the t-SNE algorithm can be found in van der Maaten and Hinton (van der Maaten and Hinton, 2008). As described in van der Maaten and Hinton (2008), the momentum term is used to reduce the number of iterations required and works best if the momentum term is small until the map points have become moderately well organized.

### Implementation

**Figure 1:**
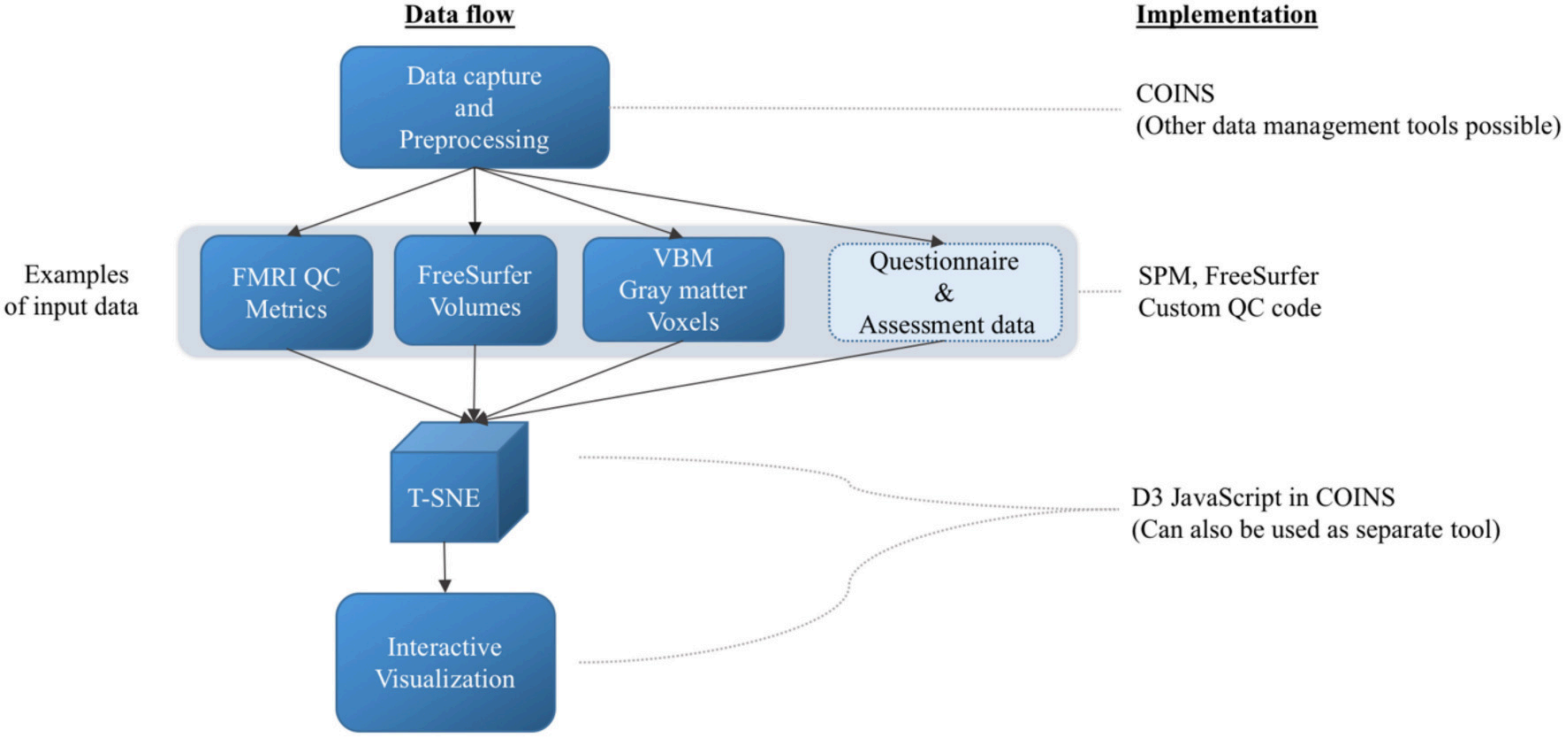
**Block diagram outlining methods and implementation**. The arrows represent the next steps.

While the approach we mention is general and can be implemented in a variety of ways, we have integrated the proposed web-based t-SNE visualization into COINS to enable easy access to tools for either prospective data currently being captured or for retrospective data uploaded after the fact (e.g., consortia created from previously collected data). This also enables us to take advantage of the large amount of data already captured within COINS with a goal of both demonstrating the feasibility of the approach and also offering such tools to existing COINS users. In addition to the COINS implementation, we also provide source code to enable implementation of the proposed approach in other tools as well. The source code is implemented in Matlab Version 7.12.0.635 (R2011a) 64-bit (glnxa64), which is a modified version of Van der Maaten's implementation freely available at https://lvdmaaten.github.io/tsne/#implementations. The block diagram shown in Figure 1 outlines the steps involved in method and implementation of the proposed technique. The questionnaire and assessment data will be used in the future works. Next we introduce 5 key use cases starting with phantom data.

### Phantom data

**Figure 2:**
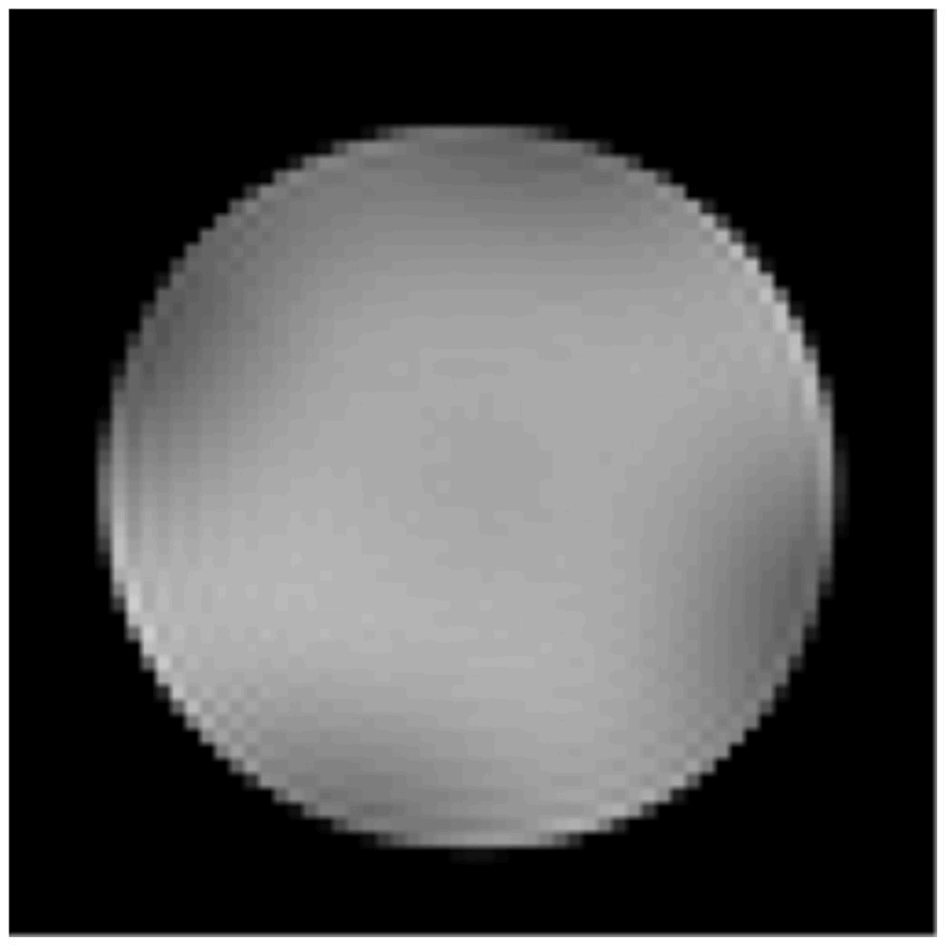
**Sample phantom scan**.

Phantom scans are typically collected at various sites to perform quality control on the scanner. In certain scenarios (e.g., study specific protocols or regular scanner QC protocols) phantom scans are acquired using a study specific protocol. Figure 2 shows the cross section of a standard phantom scan. We used phantom data from a Siemens 3T TIM Trio scanner with the following parameters: TR = 800 ms, TE = 30 ms, slice thickness = 5 mm, number of time points = 500. Numerous QC metrics can be derived from phantom scans across multiple sites (Friedman and Glover, 2006) and tracked in real time; in our case we use a few simple metrics to demonstrate the concept of our proposed approach.

#### Inputs to t-SNE

We computed the following temporal QC measures from the preprocessed data to demonstrate the potential of the proposed technique to identify inconsistencies in the phantom scan collection from specific scanner and site. For each phantom scan in the above dataset, we extract the mean 49 voxels from central 7x7 neighborhood for each of the 500 time points μ_*mid*_(*i*) where *i* ∈ {1500} and compute the mean over time $\mu_{mid}=\frac{1}{N}\Sigma_{1}^{500}\mu_{mid}\left(i\right)$, the temporal forward difference (temporal derivative) of μ_*mid*_(*i*), △μ_*mid*_(*i*) where *i* ∈ {1499}, and the mean of the temporal forward difference ${△\mu }_{mid}=\frac{1}{N}\Sigma_{1}^{499}△\mu_{mid}\left(i\right)$. These were used to compute the four following temporal QC parameters (which measure mean and maximum stability over time, and mean and maximum stability of the change in time over time).

$$\begin{array}{l} {\text{QC}}_{1}=\left\{\underset{\text{i}}{\max}\mu_{mid}\left(i\right)-\underset{\text{i}}{\min}\mu_{mid}\left(i\right)\right\}/\mu_{mid}\\ \;{\text{QC}}_{2}=\sqrt{\frac{1}{N}\sum_{1}^{500}{\left(\mu_{mid}\left(i\right)-\mu_{mid}\right)}^{2}}\\ \;{\text{QC}}_{3}=\underset{\text{i}}{\max}\Delta \mu_{mid}\left(i\right)-\underset{\text{i}}{\max}\Delta \mu_{mid}(i)\\ \;{\text{QC}}_{4}=\sqrt{\frac{1}{N}\sum_{1}^{500}{\left(\Delta \mu_{mid}\left(i\right)-\Delta \mu_{mid}\right)}^{2}} \end{array}$$

QC_1_ is a measure of the range of intensity values normalized by using the mean of the center neighborhood of the scan. QC_2_ is a measure of range of intensity values, accounting for the changes in the phantom scan from one time point to another. QC_3_ and QC_4_ are the standard deviation of the center neighborhood and the derivative of the same. The measures QC_1_ through QC_4_ were given as inputs to the t-SNE algorithm and interactive scatter plots with color coding were created to visualize the phantom datasets and identify distinct clusters of data in a visual comparison to other clusters. Table 1 lists the values of the parameters used in the t-SNE algorithm.

### Human brain data: functional MRI

The fMRI dataset included echo planar imaging data from Table 1 using a variety of protocols across multiple sites and studies. The data were automatically preprocessed including slice time correction, motion correction, and spatial normalization using the statistical parametric mapping (SPM5) toolbox (Ashburner and Friston, 2000, 2005). The following QC values are computed from these data and are given as inputs to t-SNE algorithm.

- Spatial normalization diagnostics:
  - Pearson correlation of the normalized slice time-corrected nifti image with the SPM MNI template.
  - Spearman correlation value of the normalized slice time-corrected nifti image with the SPM MNI template.

The above correlation metric that we use have been vetted on tens of thousands of data sets collected across multiple scanners and sites, and has been used in previously published work to identify outliers (Segall et al., 2012).

- Motion diagnostics:
  - Translational distance along x, y, z: *S* = (*x*^2^ + *y*^2^ + *z*^2^)^1∕2^ where *x*, *y*, and *z* are the movement (measured in millimeters) of each time point with respect to the reference, measured along the *x*, *y*, and *z* axis, respectively.
  - Framewise displacement root mean squared: $FD_{rms}=diff{\left[{pitch}^{2}+{roll}^{2}+{yaw}^{2}\right)}^{1∕2}$.
  - *pitch*, *roll*, and *yaw* are the movement (measured in radians) of each time point with respect to the reference, measured along *pitch*, *roll*, and *yaw*, respectively. Diff gives the difference between movement of each time point to its previous time point, starting from the second. The following QC metrics are computed from the translational distance and framewise displacement values calculated using the above equations.
  - mean (*S*).
  - max (*S*).
  - mean (*FD*_*rms*_).
  - max (*FD*_*rms*_).

### Human brain data: freesurfer volumes

The sMRI datasets include 3D T1-weighted scans across a variety of studies in various stages of data collection and are shown as SMRI-1 in Table 1. These data are preprocessed using FreeSurfer v. 5.3 resulting in volumetric measurements of various brain regions (Fischl, 2012). A standard FreeSurfer pipeline for surface and volume based reconstruction is used for preprocessing the data. FreeSurfer contains a fully automatic structural imaging stream for processing cross sectional and longitudinal data. It provides many anatomical analysis tools, including: representation of the cortical surface between white and gray matter, representation of the pial surface, segmentation of white matter from the rest of the brain, skull stripping, B1 bias field correction, nonlinear registration of the cortical surface of an individual with a stereotaxic atlas, labeling of regions of the cortical surface, statistical analysis of group morphometry differences, and labeling of subcortical brain structures. From this dataset, 65 volumetric measurements from various brain regions (i.e., FreeSurfer aparc regions as outlined in the Appendix D) were used as inputs to the t-SNE algorithm. The labels for these regions are outlined in Appendix C.

### Human brain data: voxel based morphometry gray matter maps

The sMRI scans from the SMRI-2 dataset, also including 3D T1-weighted pulse sequences from a variety of studies in various stages of data collection, are shown in Table 1. These data are run through a voxel based morphometry (VBM) pipeline using the SPM5 software. VBM is a neuroimaging analysis technique that allows investigation of focal differences in brain anatomy, using the statistical approach of statistical parametric mapping (Ashburner and Friston, 2000, 2005). The unmodulated gray matter concentration images from the VBM pipeline are normalized to the SPM template. The use of modulated vs. non-modulated is a choice which is made quite variably in the literature and in some cases the non-modulated maps are preferable (Meda et al., 2008). Our proposed approach can be used on either or both depending on the goals. For each scan, the voxel values at every location from all the brain slices are first summed across slices to reduce computational complexity and run time in the conceptual approach, resulting in a matrix size of 91 × 109. All the voxel values from this image from each scan are then used as inputs to t-SNE algorithm.

## Results

We review results from each of the four use cases described in the Methods Section. In addition, a demo of the t-SNE visualization is available at http://portal.mrn.org/d3vis_demo.

### Phantom data

**Figure 3:**
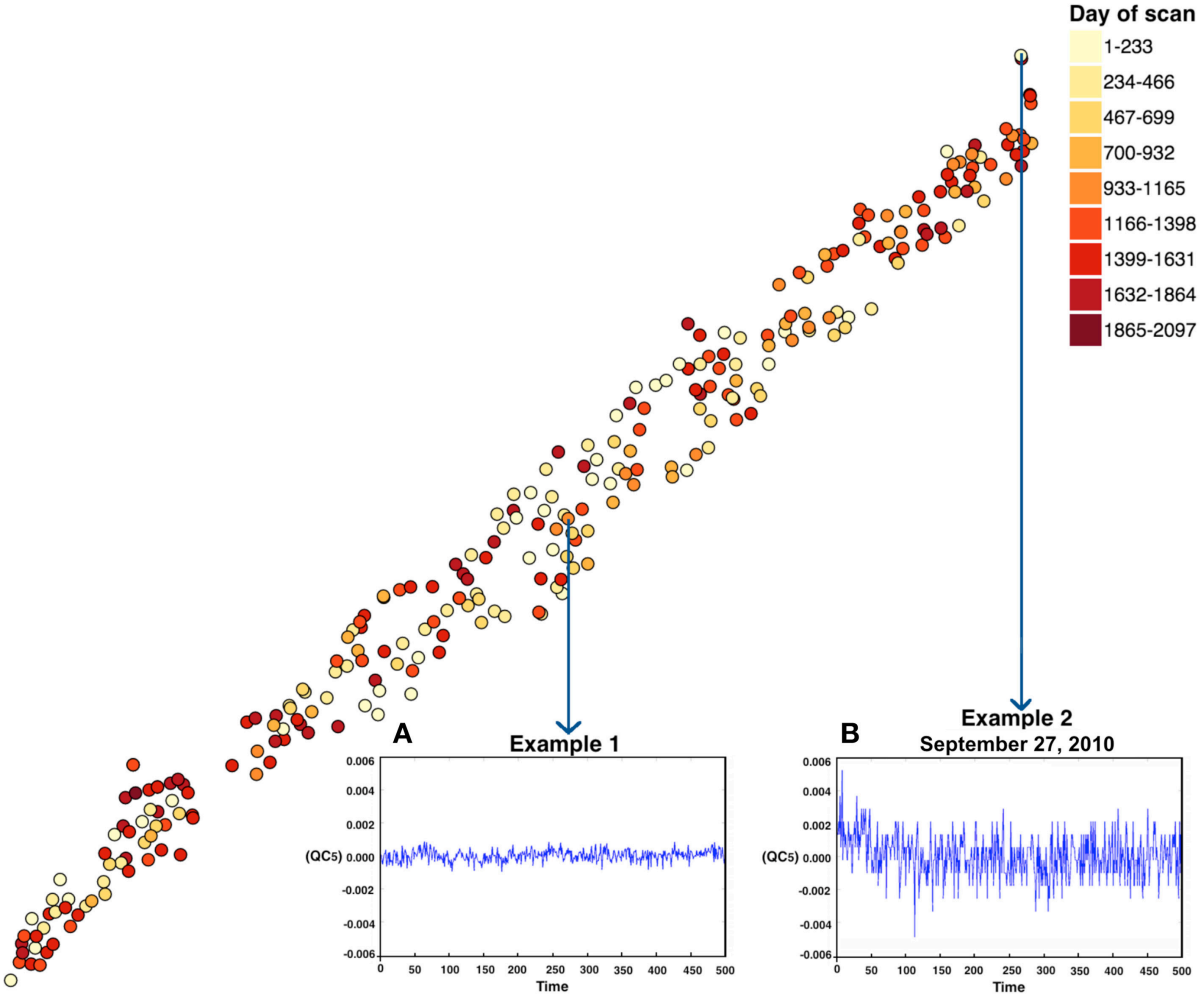
**t-SNE output for the phantom data**. Note, the X and Y coordinate on the plot are not labels as they are arbitrary and simply represent the two dimensions of the t-SNE result, one can infer that if two points are closer together in the 2D graph their data are more similar to one another as quantified by the t-SNE criteria mentioned above. Each point represents a single scan. Two representative points [**(B)** one from an extreme case representing a relatively poor QC result and **(A)** one near the middle of the points] are marked in the figure. In this case it thus appears that the “worse” QC values are in the upper right corner of the plot.

The phantom was scanned over 1868 days. t-SNE results color coded by date are shown in Figure 3. The computation time for running the t-SNE on 277 phantom scans of 4 dimensions is ~30 s. The plot shown in Figure 3B reveals that there was an inconsistency in the indicated phantom scan which was collected on September 27, 2010 at 8:56 p.m. The meta-information for each scan including the scan date is found by hovering the mouse over each scan in the plots. Figure 3 shows example time courses for the extreme phantom (Figure 3B) scan relative to a more central phantom scan (Figure 3A), which is picked arbitrarily to show the difference in the scan collection. Two key observations are that (1) the scale of change is much larger for example 2 and (2) there is clear evidence of an initial drift downward in example 2. The proposed phantom visualization tool enabled a quick summary of how any given phantom scan compares on a number of QC measures in a single 2D plot. We only used four of many possible QC measures in this plot; ideally one would incorporate spatial, temporal, and other QC measures within the analysis. Scans in the plots that are far from the majority of points or on the edge provide a guide for further exploration that can readily be followed up. Such an approach can help establish a consistent data collection environment over a large period of time across various scanners, sites, hardware upgrades and collection protocols.

### Human brain data: functional MRI

**Figure 4:**
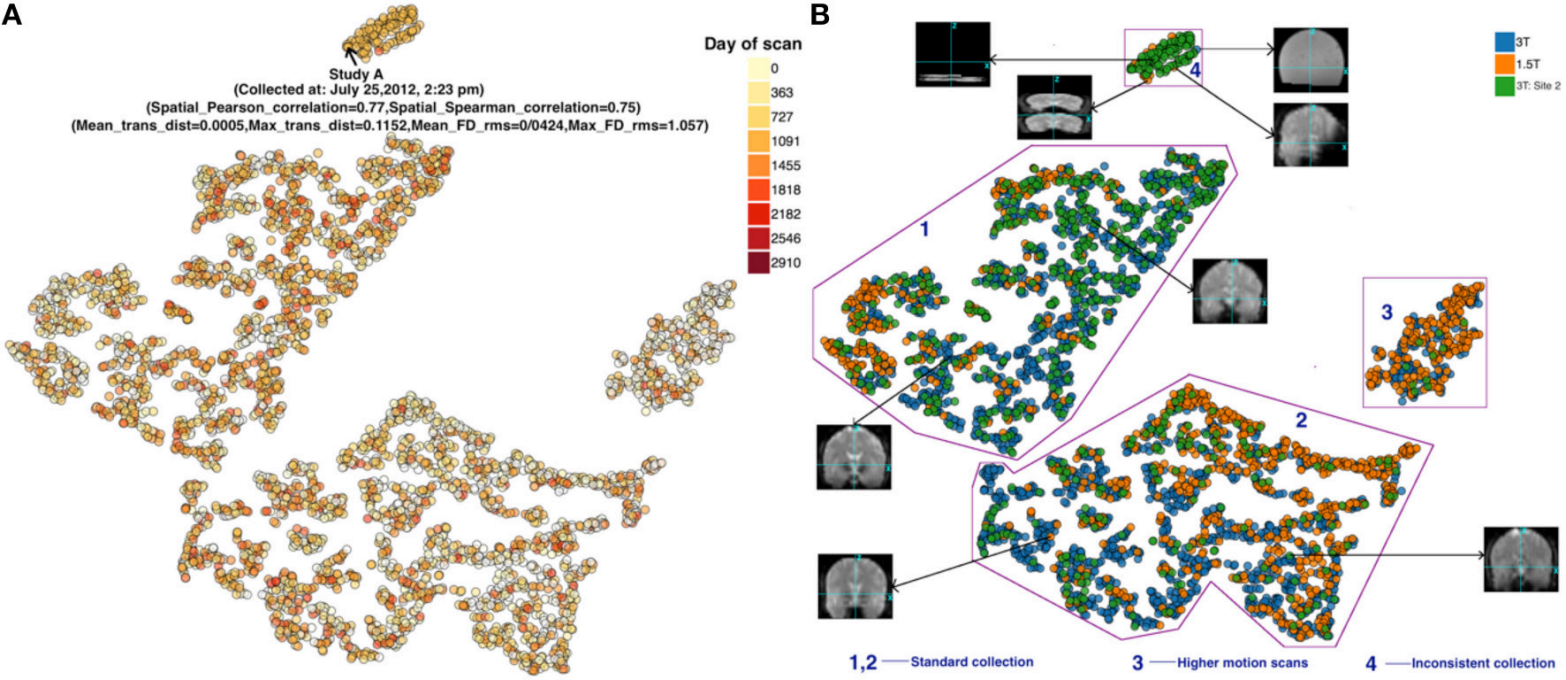
**(A)** FMRI t-SNE scatter plot-color coded by (left) date of scan, and **(B)** (right) scanner type. Groups 1 and 2 both appeared to include good quality scans but were farther away from the majority of points than the other groups. Group 3 demonstrated high motion. Group 4 scans were noticeably different compared to scans outside these groups (see example thumbnails in figure). Note, the X and Y coordinate on the plot are not labels as they are arbitrary and simply represent the two dimensions of the t-SNE result, one can infer that if two points are closer together in the 2D graph their data are more similar to one another as quantified by the t-SNE criteria mentioned above.

The compilation time for running the t-SNE on 3910 datasets of six dimensions each is ~10 min on a computer with the configuration detailed in Table 1. Figures 4A,B depicts the resulting plot color coded by scan date and site/scanner, respectively. One of the advantages of our D3 implementation, which helps enhance the usability of our proposed approach, is the interactivity of the available information. For example, Figure 4A (left) shows additional meta-data that are displayed dynamically by the D3 JavaScript interface within the web browser. Based on the provided input data, information about each data point in the plot is displayed by hovering over the dots. This helps in identifying the reason behind grouping of the data with respect to study, scan date, type of scan and values of QC measures and one can also use this to “drill down” to the input data.

Figure 4B reveals that the scans are grouped well according to their input QC measures. This was verified by checking the QC values of various scans in real time on the web page. Four somewhat visually distinct groups are identified in the Figure 4B (right). Several interesting observations about these groups are worth pointing out.

First, group 3 and 4 were farther away from the majority of data points than the rest. Group 3 is comprised of scans which had high motion relative to the scans outside of this group. These scans had relatively high value of maximum translation distance and maximum framewise displacement, compared to the scans in groups 1, 2, and 4. A majority of the scans in this group were from the 1.5T scanner. A closer look at the scans in group 4 revealed that they are also noticeably different (and of poor quality) compared to scans outside these groups. The majority of the scans in this group are from the second 3T dataset from a specific study with a very different acquisition protocol than the other studies. All the 120 scans from this study (group 4) were acquired between July 17, 2012 and July 27, 2012. This type of information might be useful during data collection of a new study, e.g., to adjust parameters or evaluate consistency across various studies. Importantly, two groups (3 and 4) captured high motion and noticeably poor quality scans, respectively. Groups 1 and 2 both appeared to be of good quality. Likely with additional QC measures (incorporating both spatial and temporal measures) the results will be even more informative.

**Figure 5:**
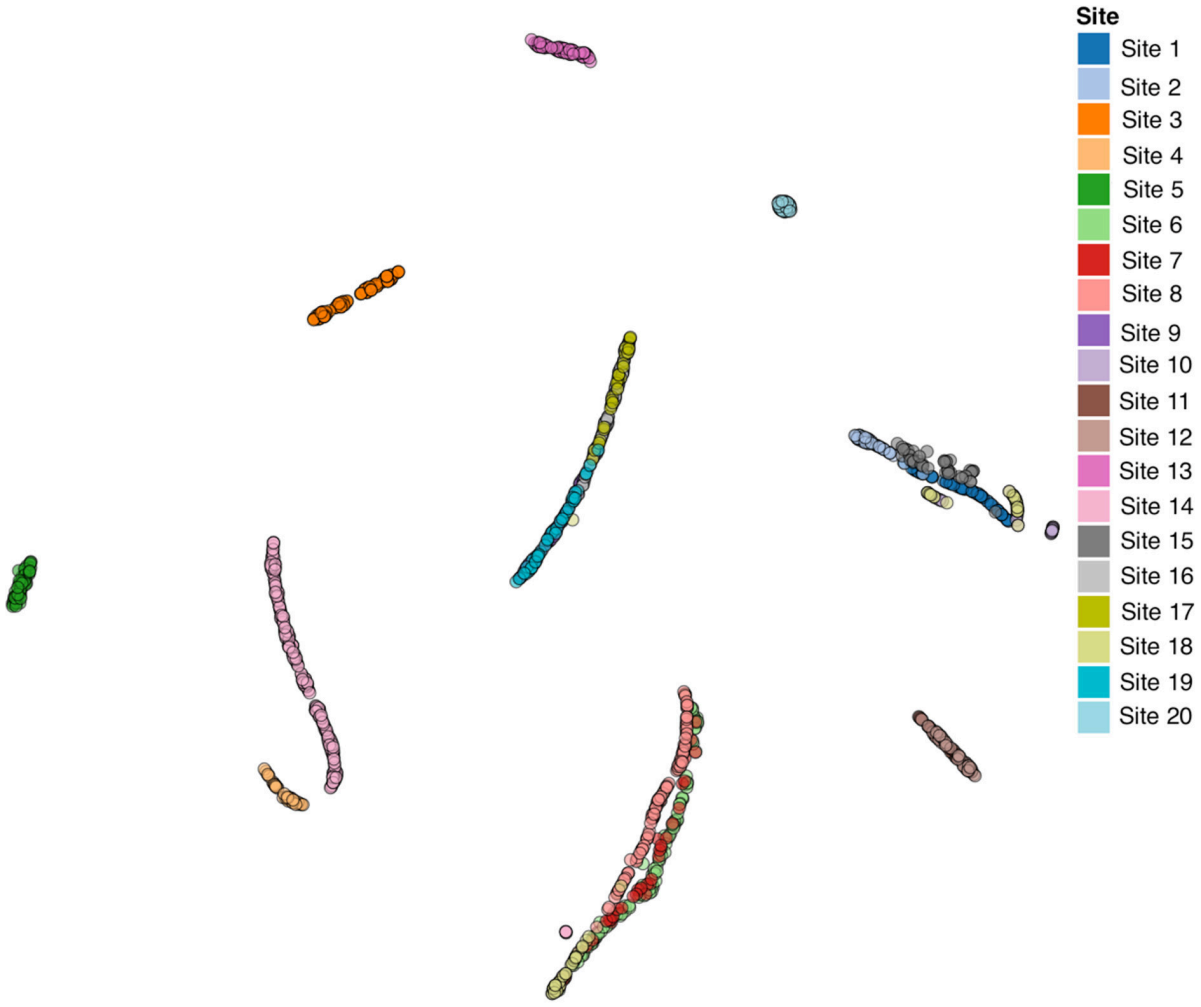
**ABIDE fMRI Data**. Note, the X and Y coordinate on the plot are not labels as they are arbitrary and simply represent the two dimensions of the t-SNE result, one can infer that if two points are closer together in the 2D graph their data are more similar to one another as quantified by the t-SNE criteria mentioned above.

We also performed a similar analysis of the autism brain imaging data exchange (ABIDE) resting fMRI data, which is available via the COINS data exchange (http://coins.mrn.org/dx). The ABIDE data has been pre-processed and multiple spatial and temporal QC measures are provided at https://github.com/preprocessed-connectomes-project/quality-assessment-protocol/tree/master/normative_data. This dataset is labeled ABIDE fMRI in Table 1. We incorporated both the spatial and temporal QC measures into a t-SNE analysis. Results are reported in Figure 5 and are color coded by site. In this case, the data were collected in a heterogeneous (non-coordinated) manner and it is clear that the QC measures are considerably more visually distinct and highly correlated with the site of data collection (though some of the sites do group together tightly). We also evaluated results by scanner type and vendor, while this was highly related to site, it was clear that it was not the scanner type that was the primary driver in the similarity. We speculate that the largest source of variation is likely due to the specific instructions and data collection protocol that was followed for the study. While we were not able to assess in this study, it would be very interesting to understand what about the data collection resulted in different sites being more or less similar. This could aid in planning future studies.

These initial results provide a proof-of-concept that a lower dimensional visualization approach like t-SNE has the potential to provide useful groupings of fMRI data based on QC metrics. Next we summarize results from two different sMRI use cases. In both of these cases, instead of utilizing QC metrics we use values based on volumetric measurements of various brain regions or gray matter concentration, in order to demonstrate the potential of our proposed approach to visualize these data along various dimensions.

### Human brain data: freesurfer volumes

**Figure 6:**
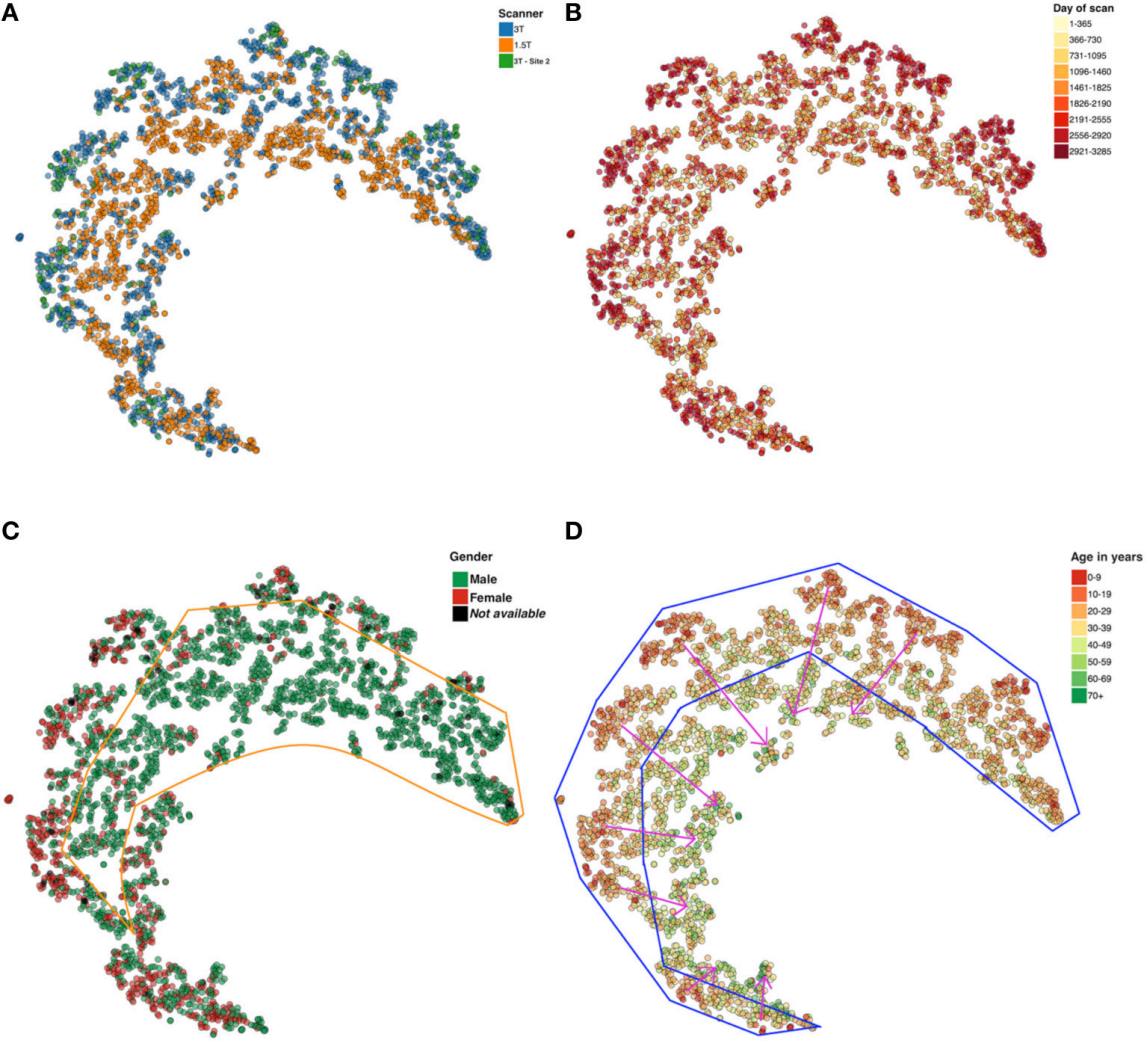
**t-SNE plots color coded by (A) Scanner type (B) Day of scan (C) Gender, and (D) Age**. Note, the X and Y coordinate on the plot are not labels as they are arbitrary and simply represent the two dimensions of the t-SNE result, one can infer that if two points are closer together in the 2D graph their data are more similar to one another as quantified by the t-SNE criteria mentioned above.

The first sMRI use case involves t-SNE visualization of FreeSurfer volumetric values. The compilation time for running t-SNE on 2806 datasets of 65 dimensions each is ~20 min. Using the resulting t-SNE scatter plot, we color code along four different categories (each of which is available in a dropdown box from the browser) as shown in Figure 6. Figure 6A is color coded by the three different scanners included in the data set. In the scatter plot shown in Figure 6B, every day from the first scan to the last scan in the dataset is given a color on the heat map for easy visualization of scans that are collected in the similar period of time. Note that most of the more recent scans are on the “outside” rim of the partial circle of data. Figure 6C shows that the males and females are grouped in different regions in the plot. In Figure 6D results are presented as a function of subject age. As shown in Figure 6D, the oldest subject in the dataset is 88 years old, the mean age is 32.81 years and the standard deviation is 12.86. Results show a visible trend in the way, the younger to older population are grouped in the plot; younger subjects are on the periphery and older subjects toward the center (as indicated by the pink arrows).

### Human brain data: voxel based morphometry gray matter maps

**Figure 7:**
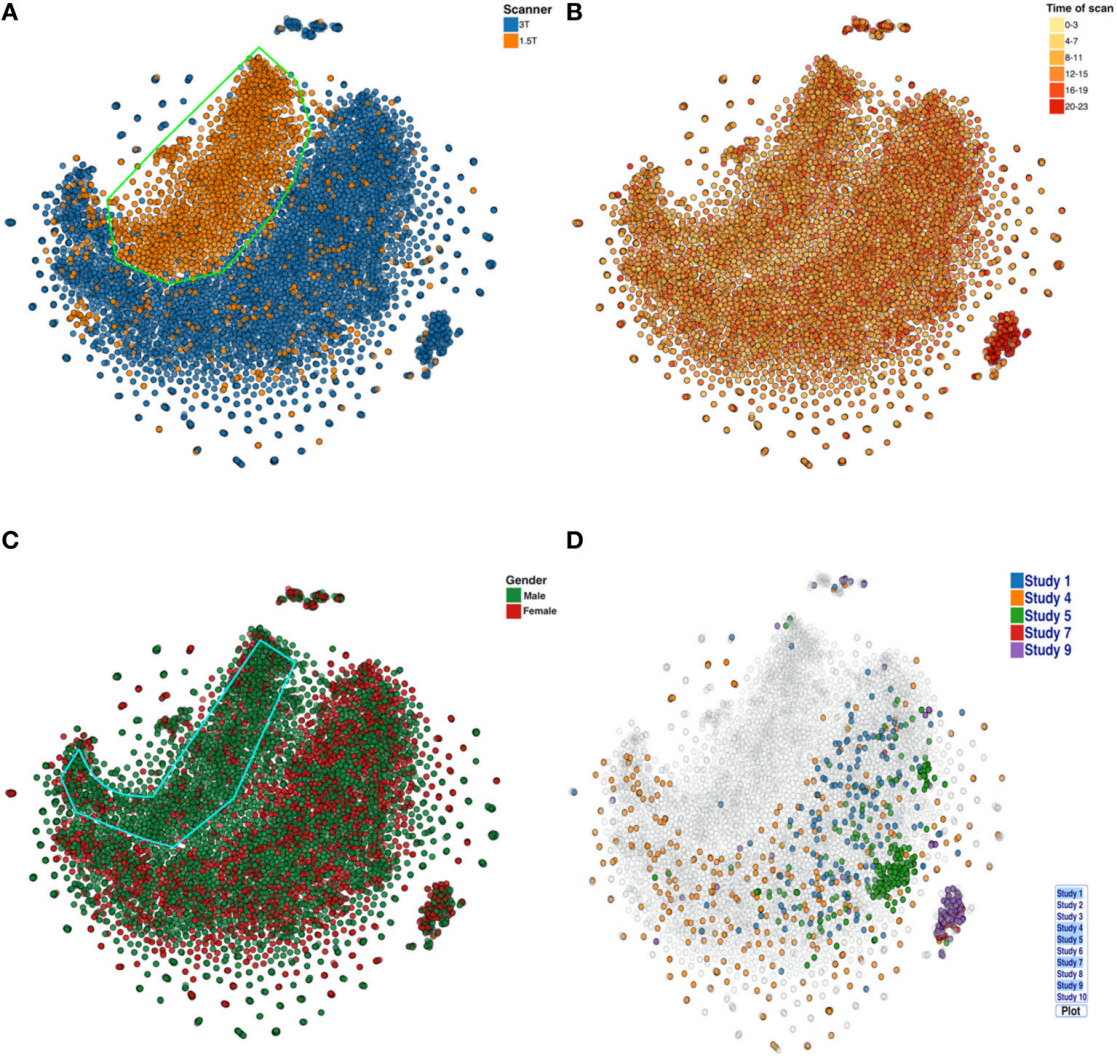
**t-SNE plots color coded by (A) Scanner type (B) Scan acquisition time (C) Gender, and (D) Studies**. Note, the X and Y coordinate on the plot are not labels as they are arbitrary and simply represent the two dimensions of the t-SNE result, one can infer that if two points are closer together in the 2D graph their data are more similar to one another as quantified by the t-SNE criteria mentioned above.

**Figure A7:**
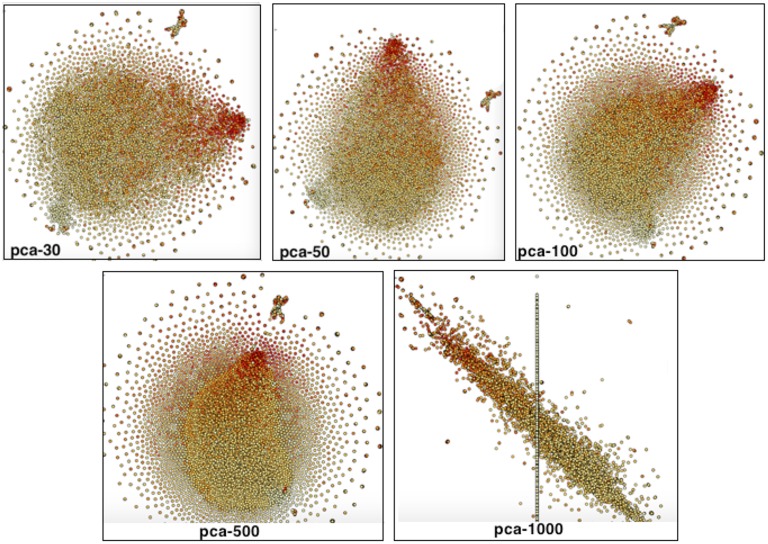
**sMRI-2 PCA experiments**. Single gray matter slice from each subject in the data set was used in these experiments. Plots are color coded by age. Note, the X and Y coordinate on the plot are not labels as they are arbitrary and simply represent the two dimensions of the t-SNE result, one can infer that if two points are closer together in the 2D graph their data are more similar to one another as quantified by the t-SNE criteria mentioned above.

In the last use case, voxelwise (reduced) VBM results are analyzed with t-SNE. Because running the t-SNE on 9963 datasets of 9919 dimensions each takes ~7 h, real-time computation of the plots is not practical. Nonetheless, it is still possible to calculate a new plot daily. Color coding of various categories (as in the four plots below) is not computationally expensive and can be done dynamically within the browser based on user selection. The plot in Figure 7A reveals that the structural scans collected from the 1.5T scanner are systematically different compared to scans collected on the 3T scanner. Both show a similar spiral-like shape, but they are mostly not overlapping. Figure 7B is color coded by time of day when the scan was collected (this was in part done due to the recent finding that time of day was associated with brain volume; Nakamura et al., 2015). In our cases, the data did not obviously support such a conclusion. Figure 7C shows that majority of the scans from the 1.5T data are from males. Figure 7D shows the scans from a few selected studies. Using the proposed visualization tool, the user can select specific studies from a list and potentially identify interesting patterns in the data across studies of interest. This information helps us recognize and estimate the effect of the type of scanner, scanning protocol, study, and other variables on the data. Note, we also compared results for several PCA settings and they were similar (see Figure A7).

**Figure 8:**
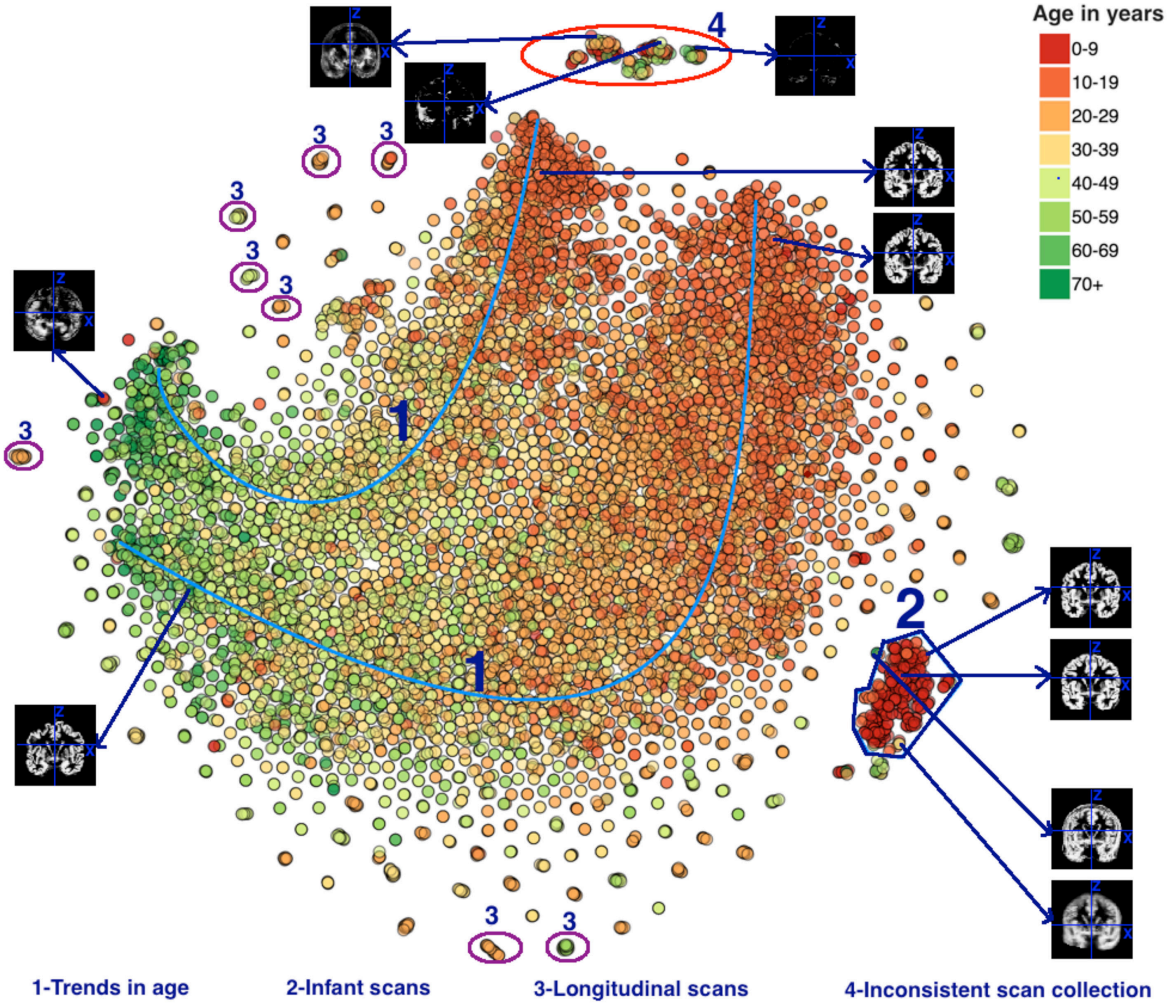
**t-SNE plot color coded by age**. Groups 1–4 were visually identified and highlighted based on interesting observation and are discussed in the text. Note, the X and Y coordinate on the plot are not labels as they are arbitrary and simply represent the two dimensions of the t-SNE result, one can infer that if two points are closer together in the 2D graph their data are more similar to one another as quantified by the t-SNE criteria mentioned above.

In Figure 8 the data are colored by age, revealing some interesting results. The oldest subject in the dataset is 89 years old, the mean age is 30.95 years and the standard deviation is 15.02 years. Four groups were visually identified in Figure 8 and several interesting patterns were found in those datasets. First, this plot reveals an interesting trend in the scans collected from older population to the younger population. This trend is marked as 1 in Figure 8. Note that both the 1.5 and 3T data, though mostly not overlapping, are showing the same trend from old to young. We also see that the younger subjects have more variability (scatter) in the plot than the older subjects. The scans in group 2 are from an infant population and are grouped in a tight group indicating that the t-SNE algorithm grouped similar datasets efficiently. The scans in group 3 are the scans from the same subject who appeared multiple times under the same or different studies. Scans from the same subject scanned multiple times have nearly identical location on the plot. Next, for group 4 we observed that the majority of the scans were collected more recently relative to the scans collected outside of this group. After examining the scans, it was noted that the gray matter maps of these scans deviated significantly from the data outside group 4 in a way that suggested a problematic segmentation (see example insets in the figure). Finally, we noticed that some scans from younger population are grouped with the scans of the older population and vice versa. Some of these scans are shown in Figure 8, highlighting the fact that their scans deviated from the other data and suggest a problematic segmentation or input data. These populations could be interesting to study as they indicate a unique structural pattern in the brain visually compared to the population in their age demographics, such as older individuals who show less or greater reductions in brain volume compared to their age-specific cohort.

## Discussion

Using a novel visualization technique, we extracted meaningful information from large, neuroimaging data sets. This technique has great potential in enhancing quality control for large data sets and enables enhanced pattern identification across data sets coming from a diverse set of sites, scanners, and participant demographics. It can be used to help identify “low quality” data sets or to identify data sets that require additional processing. In addition, it can be used to identify interesting patterns with variables of interest such as age, gender, or diagnosis.

These methods build on previous work in large-scale neuroimaging developments, including comparisons across scanners and equipment, and quality assurance metrics for multi-scanner studies (Gunter et al., 2009; Glover et al., 2012; Chen et al., 2014; Turner, 2014). This platform now leverages those approaches and resulting large datasets for a more dynamic look at trends across various neuroimaging measures. As large neuroinformatics resources become more common, the ability to quickly perform these large comparisons becomes a valuable tool to assist with identification of outliers in the data sets. The availability of large datasets has led to recommendations of different methods for what qualifies a data set as unusable. For example, Yan et al. (2013), who used large, publicly available resting state fMRI datasets to compare different movement correction techniques within different analyses. By examining the commonalities and differences across these methods they were able to develop more general and also nuanced recommendations (Yan et al., 2013). Currently, the threshold beyond which data are not usable depends on the analysis being done and the comparability of the remaining data. Being able to rapidly visually compare datasets across a range of QC measures enables more targeted identification of unusable data, by identifying datapoints that are outliers not on any single QC measure but across a number of them.

A related project includes INVIZIAN (Bowman et al., 2012), which clusters large samples of brain measures based on similarity, and allowed visualization for a more informed, detailed comparison of a given subject's data against the others. Existing approaches can be found at qc.loni.usc.edu and www.neugrid4you.eu, though these are primarily designed as QC feedback approaches rather than interactive visualization and comparison. The COINS visualization platform is intended for large data visualization at a glance, rather than more detailed inter-subject comparisons. Our approach, and those enabling finer-grained comparisons would work well in tandem, with the combination of multiple visualizations at different scales affording both rapid subtyping, and detailed comparisons for more specific hypotheses. Another recent project found meaningful clusters using multimodal imaging data (Mwangi et al., 2014). Another recent example showed a relationship of measures based on gray matter segmentation with Huntington's disease severity in which the output of a deep learning analysis was visualized using tSNE (Plis et al., 2014). This approach, combined with the multiple examples in the current study, suggest a promising future for such visualization of patterns of regularities related to brain data. Future studies should examine whether diagnostic categories can be meaningfully captured (or refined into subcategories by using the multi-dimensional dimension reduction approach employed here). A More generally neurophenotyping efforts may benefit from such approaches.

### Limitations and future work

There are several limitations of the current work. First, we have incorporated only a limited number of QC metrics in order to demonstrate the utility and feasibility of the approach. Certainly for large-scale use, the number of metrics should be increased, and the impact of the incorporated metrics should be systematically evaluated. In addition, the t-SNE approach we use is only one possible approach to visualization. The choice of t-SNE parameters is data dependent, and thus more work needs to be done to determine these parameters, however in our experience, the overall patterns were robust on a range of parameter choices. In general, we suggest providing a tunable interface so end users can adjust the parameters as desired. Future iterations could further optimize the code and potentially increase the computational capacity to reduce the run time of the t-SNE algorithm. And finally, the relationship between the number of subjects and the number of input parameters should be further explored to identify the potentially limitations. There is a growing need for approaches like the one we present, as large shared datasets and multi-site studies are increasing. The current study clearly shows the potential for such an approach. There are numerous other applications, including comparison of different processing pipelines, or visualization of data that was processed differently. Many studies currently include visualization for specific project or goal (Fox et al., 2011; Romano et al., 2014). What we are proposing is a general visualization tool that can provide information about the data “as it arrives” with a goal of bringing the investigator closer to the data to capture unanticipated patterns. Our visualization approach can also be applied to questionnaire or clinical data as well. Decentralized processing approaches, including those that incorporate additional privacy protection, can also benefit greatly from such tools (Sarwate et al., 2014; Carter et al., 2015). Automated approaches for identifying relevant clusters will also be important going forward, but are not the focus of this work. In conclusion, we believe this work shows a promising future directly that needs to expand dramatically going forward.

## Conclusions

The results from the proposed technique show the potential benefit of using 2D visualization to explore high-dimensional brain imaging QC metrics or variables of interest. As neuroimaging data collection methods expand and standardization becomes difficult, this visualization technique provides an easy way to perform quality control and find interesting patterns in datasets across multiple sites, studies, and other variables of interest. In future work, we plan to apply this technique to larger datasets and datasets outside the field of neuroscience as well as evaluate the utility of 3D visualization approaches for improving the grouping of datasets. In addition, it may have utility in identifying “biomarkers” of different neurological or psychiatric disorders.

## Author contributions

SRP, VC, SMP conceived of the project. SRP performed the analysis and wrote the paper. RW coded the demo site and contributed to the analysis. All the authors helped interpret the data/edited/revised the manuscript.

## Funding

The work was in part funded by NIH via a COBRE grant P20GM103472 and grants R01EB005846, 1R01EB006841, 1R01DA040487 as well as NSF 1539067.

### Conflict of interest statement

The authors declare that the research was conducted in the absence of any commercial or financial relationships that could be construed as a potential conflict of interest.

## Figures and Tables

**Figure A1:**
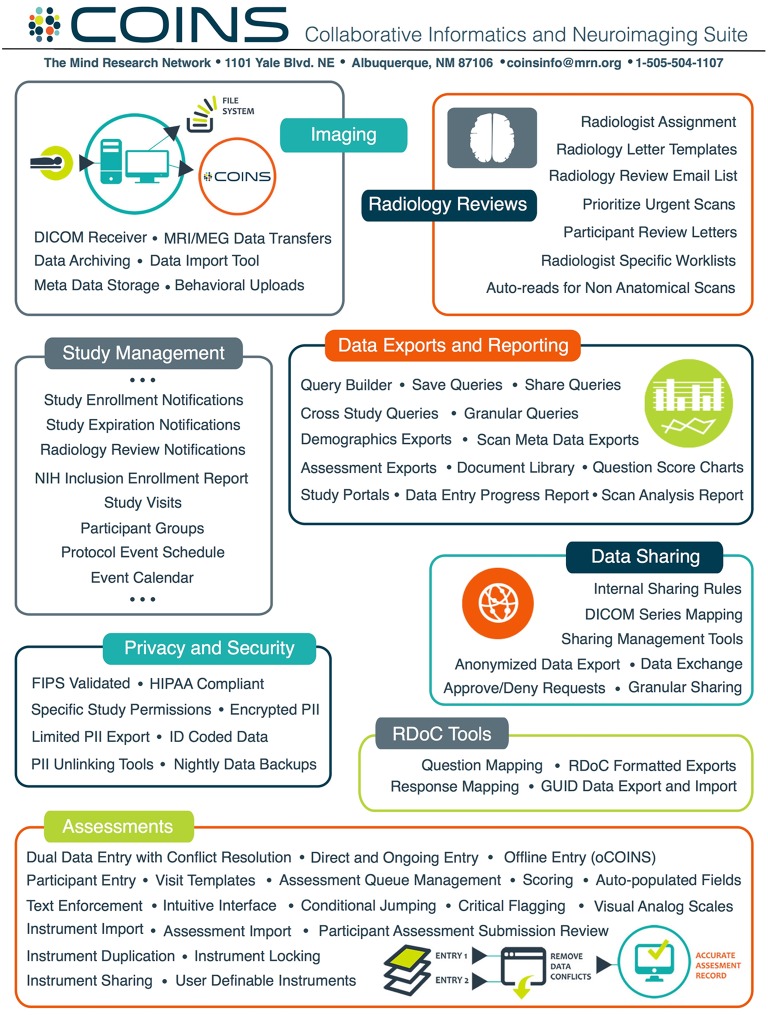
**A high-level overview of COINS Features**. COINS provides a full suite of tools including image and assessment capture, study management, security, offline data capture, and data export and reporting. For more information about COINS, see http://coins.mrn.org and previous publications (Scott et al., 2011; King et al., 2014; Wood et al., 2014)^2^.

**Figure A2:**
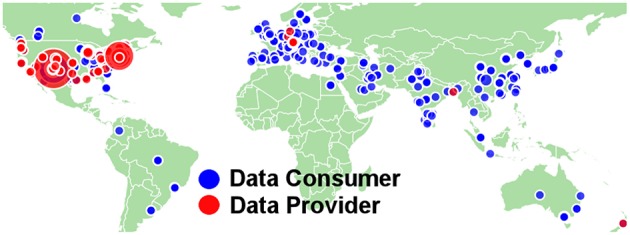
**COINS data consumers and providers (34 states and 42 countries)**.

**Figure A3:**
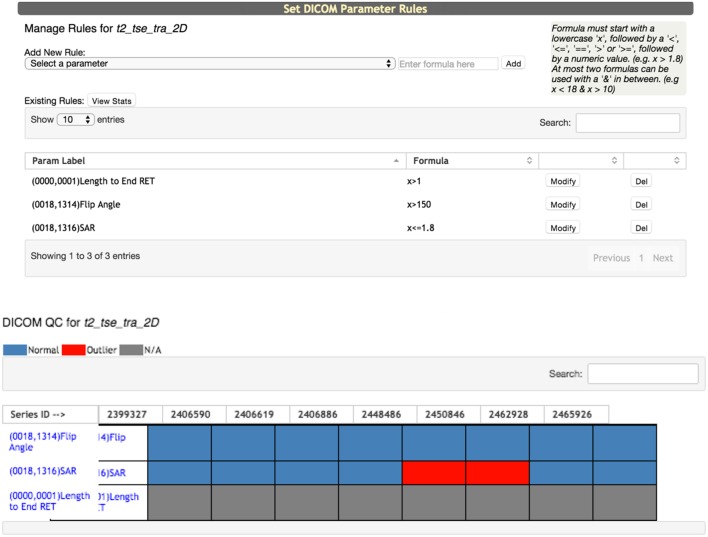
**DICOM QC tool enables selection of one or more dicom parameters for tracking**.

## Footnotes

^1^d3, http://d3js.org.

## Appendix A: COINS implementation

The core infrastructure onto which we demonstrate the proposed QC approach is built is COINS (http://coins.mrn.org; Scott et al., 2011), a mature end-to-end system for data capture and study management (King et al., 2014), archiving, and sharing (Wood et al., 2014), with a large variety of available features (see Figure A1). COINS currently serves multiple investigators and imaging centers world-wide (see Figure A2; Bockholt et al., 2009; Scott et al., 2011; King et al., 2014; Wood et al., 2014). COINS includes a set of tools that enables study specific automated and manual capture and archiving of phantom or human DICOM data in an organized manner in addition to an extensive suite of non-imaging data visualization tools. The COINS system also provide a dashboard tool to visualize summary metrics on demand. COINS can handle a variety of imaging modalities and analysis tools, and includes data capture and archival services that automate the transfer, organization, backup and processing of imaging data directly from the MRI scanner. COINS was used to upload/share data for the CoRR consortium consisting of over 4000 rest fMRI datasets (Zuo et al., 2014) as well as the enhanced NKI Rockland dataset (1000 participants with extensive phenotyping)^3^. COINS fully integrates offline and online handling of multi-site study management, radiology reading, assessments including advanced question types (King et al., 2014), DICOM image capture, and automated processing (Wood et al., 2014). COINS is also a fully hardened multi-site tool, with site-specific management tools, a range of secure user roles and encryption. Containing over 40,000+ imaging sessions from 33,000+ participants and 440,000+ assessments, COINS has undergone substantial testing and continues to grow. COINS tools assimilate and validate data automatically and incorporate tools for automated range checking and scoring (Scott et al., 2011; King et al., 2012).

One key aspect of COINS is the incorporation of multiple quality assurance (QA) and QC tools. One example of this is for non-imaging data including tools such as range-checking of question responses, automated queuing of questionnaires, and automated scoring (King et al., 2014). Regarding imaging data examples of such tools include automated DICOM archiving by study, and the DICOM QC tool which enables instant checking of DICOM parameters to identify deviations in a given protocol as soon as possible (see Figure A3).

## Appendix B: parameters used for tSNE

**Table:**

Maximum number of Iterations	2000
Initial momentum	0.5
Final momentum	0.8
Iteration at which momentum is changed	250

## Appendix C: summary of data

**Table:**

**FMRI dataset**			
**Site**	**Scanner**	**# Studies**	**# Scans**
1	Siemens 3T Triotim	37	2183
1	1.5T	3	1022
2	Siemens 3T Triotim	22	705
			Total=3910

**Table:**

**sMRI dataset from FreeSurfer v. 5.3**			
**Site**	**Scanner**	**# Studies**	**# Scans**
1	Siemens 3T Triotim	26	1443
1	Siemens 1.5T Avanto	8	1143
2	Siemens 3T Triotim	12	220
			Total=2806

**Table:**

**sMRI Gray matter images whole brain dataset**			
**Site**	**Scanner**	**# Studies**	**# Scans**
Mind Research Network	Siemens 3T Triotim	121	7232
Mind Research Network	Siemens 1.5T Avanto	9	2731
			Total=9963

## Appendix D: freesurfer labels used for sMRI data

Left-Lateral-Ventricle

Left-Inf-Lat-Vent

Left-Cerebellum-White-Matter

Left-Cerebellum-Cortex

Left-Thalamus-Proper

Left-Caudate

Left-Putamen

Left-Pallidum

3rd-Ventricle

4th-Ventricle

Brain-Stem

Left-Hippocampus

Left-Amygdala

Cerebrospinal Fluid

Left-Accumbens-area

Left-VentralDC

Left-vessel

Left-choroid-plexus

Right-Lateral-Ventricle

Right-Inf-Lat-Vent

Right-Cerebellum-White-Matter

Right-Cerebellum-Cortex

Right-Thalamus-Proper

Right-Caudate

Right-Putamen

Right-Pallidum

Right-Hippocampus

Right-Amygdala

Right-Accumbens-area

Right-VentralDC

Right-vessel

Right-choroid-plexus

5th-Ventricle

WM-hypointensities

Left-WM-hypointensities

Right-WM-hypointensities

non-WM-hypointensities

Left-non-WM-hypointensities

Right-non-WM-hypointensities

Optic-Chiasm

CC_Posterior

CC_Mid_Posterior

CC_Central

CC_Mid_Anterior

CC_Anterior

BrainSegVol

BrainSegVolNotVent

BrainSegVolNotVentSurf

lhCortexVol

rhCortexVol

CortexVol

lhCorticalWhiteMatterVol

rhCorticalWhiteMatterVol

CorticalWhiteMatterVol

SubCortGrayVol

TotalGrayVol

SupraTentorialVol

SupraTentorialVolNotVent

SupraTentorialVolNotVentVox

MaskVol

BrainSegVol-to-eTIV

MaskVol-to-eTIV

lhSurfaceHoles

rhSurfaceHoles

SurfaceHoles
